# Effect of remimazolam tosilate versus etomidate on hemodynamics in patients undergoing valve replacement surgery: study protocol for a randomized controlled trial

**DOI:** 10.1186/s13063-022-06962-x

**Published:** 2022-12-12

**Authors:** Bailong Hu, Haiyan Zhou, Xiaohua Zou, Li Tan, Tao Song, Lucas Zellmer, Xingyu Li

**Affiliations:** 1 Department of Anesthesiology, The Affiliated Hospital of Guizhou Medical University, 28 Guiyi Rd, Guiyang, Guizhou Province 550004 People’s Republic of China; 2 Department of Cardiology, The Affiliated Hospital of Guizhou Medical University, 28 Guiyi Rd, Guiyang, Guizhou Province 550004 People’s Republic of China; 3 College of Anesthesiology, Guizhou Medical University, 9 Beijing Rd, Guiyang, 550004 Guizhou Province People’s Republic of China; 4 Department of Medicine, Hennepin County Medical Center, 730 South 8th St, Minneapolis, MN 55415 USA

**Keywords:** Remimazolam tosilate, Etomidate, Hemodynamics, Valve replacement surgery

## Abstract

**Background:**

Patients with a history of cardiac disease are prone to develop cardiovascular adverse events such as hypotension, hypertension, and tachycardia during anesthesia induction. Therefore, hemodynamic stability is one of the most important concerns for induction of anesthesia in patients undergoing cardiac surgery. Remimazolam tosilate is a new, ultra-short-acting benzodiazepine agent, with the advantages of rapid onset, rapid offset, and minimal cardiorespiratory depression. We aim to compare the effect of remimazolam tosilate and etomidate on hemodynamics during anesthesia induction in patients undergoing valve replacement surgery.

**Methods/design:**

The trial is a prospective, randomized, double-blinded, controlled, single-center trial to compare the effect of remimazolam tosilate and etomidate on hemodynamics in patients undergoing valve replacement surgery. One hundred seventeen patients undergoing selective valve replacement surgery between January 1, 2022, and December 31, 2023, will be enrolled and randomly allocated into one of three groups: low-dose remimazolam group (Group LR), high-dose remimazolam group (Group HR), or etomidate group (Group E). The primary outcome is hemodynamic fluctuations during anesthesia induction (the difference between mean arterial pressure [MAP] to baseline, ▴MAP; and the difference between maximum or minimum heart rate [HR] and baseline, ▴HR). Secondary outcomes include the incidence of adverse cardiovascular events (hypotension, severe bradycardia, hypertension, tachycardia, and arrhythmia), the cumulative doses of vasoactive drugs used per patient, incidence and degree of injection pain and myoclonus, blood glucose values, and vital signs at different time points.

**Discussion:**

This research will determine the effectiveness and safety of remimazolam tosilate induction on hemodynamics in patients undergoing valve replacement surgery.

**Trial registration:**

www.chictr.org.cn identifier ChiCTR2100052535. Registered on 17th Dec 2021, http://www.chictr.org.cn/).

## Background

Patients undergoing cardiac surgery are at risk of developing hemodynamic instability, including hypotension and arrhythmia, especially during the induction of anesthesia. Etomidate is a non-barbiturate hypnotic agent that exerts a sedative and anesthetic effect through γ-aminobutyric acid subtype A (GABA_A_) receptors in the central nervous system [1]. Previous studies have shown that etomidate has minimal side effects on the cardiovascular and respiratory systems [2, 3]. Due to the low risk of hypotension, etomidate is considered to be a relatively safer agent for the induction of anesthesia in patients undergoing cardiac surgery [4, 5]. However, etomidate may result in adrenal cortex function inhibition [6, 7], nausea and vomitin g[8], injection pain [9, 10], and myoclonic movement [11, 12], which have limited its clinical application to a certain extent. Remimazolam tosilate is a new-type benzodiazepine agent, acting on the GABA_A_ receptor, which has been reported to have the advantages of rapid onset, short maintenance and recovery times, and less cardiorespiratory depression [13–15]. Therefore, we hypothesized that remimazolam tosilate may be a relatively safer agent for anesthesia induction in patients undergoing cardiac surgery. Currently, research on the comparison of remimazolam tosilate and etomidate on hemodynamics during anesthesia induction in patients undergoing cardiac surgery is lacking. The aim of this study is to compare remimazolam tosilate and etomidate on the hemodynamics in patients undergoing valve replacement surgery.

## Methods

### Trial design

**Fig. 1:**
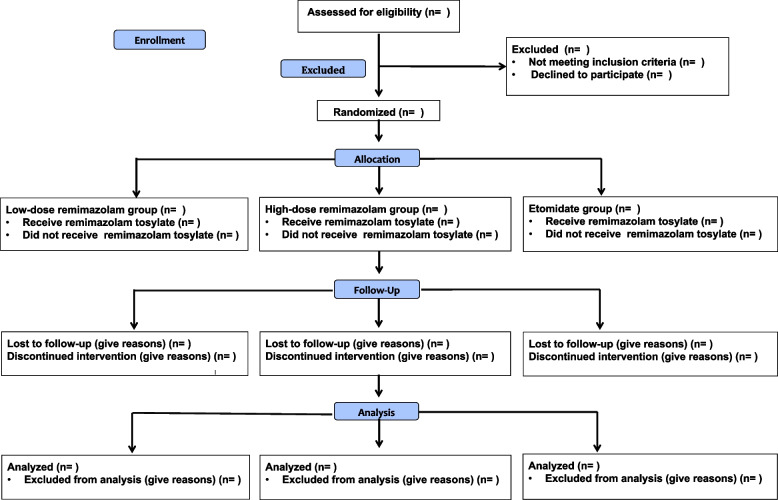
Consort flow diagram of the trial design

The trial is a prospective, randomized, controlled, double-blinded, single-center trial designed to evaluate the effect of remimazolam anesthesia induction on hemodynamics in patients undergoing valve replacement surgery. Patients will be enrolled and randomly allocated into one of the three groups: low-dose remimazolam group (Group LR), high-dose remimazolam group (Group HR), or etomidate group (Group E). The trial scheme is illustrated in Fig. 1.

### Ethics

This study protocol has been approved by the Chinese Ethics Committee of Registering Clinical Trials (Ref: ChiECRCT20210524) and registered on www.chictr.org.cn (ChiCTR2100052535). This trial will be performed according to the principles of the Declaration of Helsinki (Edinburgh 2000 version). Written informed consent will be obtained from each patient before enrollment.

### Sequence generation

All eligible participants will be randomized to one of the three groups (Group LR, Group HR, or Group E ) using computer-generated random numbers and a 1:1:1 allocation ratio. The randomization assignment will be generated by one independent investigator using a computer-generated random number sequence.

### Allocation concealment mechanism

Numbered-Opaque-sealed envelopes will be used to conceal the allocation information. The nurse who is blinded to the trial will open the opaque-sealed envelopes after the participant’s arrival to the operation room. The opaque-sealed envelopes will be stored in a secure place throughout the study. The operator, anesthesiologists, and outcome investigators will be blind to the allocation information.

### Blinding

The patients, attending anesthesiologist, and nurse, as well as the researchers, will not be aware of the randomization and allocation concealment throughout the study. All syringes containing the experimental medications will be covered with masking tape to conceal the anesthetic information. Data will be analyzed and collected by an investigator who is blinded to the patient’s group allocation.

### Participants

#### Study population

Patients who are scheduled to undergo elective valve replacement surgery aged 18–65 years old will be recruited from the Affiliated Hospital of Guizhou Medical University. The trial will be ongoing from January 2022 to December 2023.

##### Inclusion criteria

1. American Society of Anesthesiologists (ASA) Physical Status I–III.
2. Patient aged between 18 and 65 years old.

##### Exclusion criteria

1. Patients who are allergic to or possess contraindications to benzodiazepines, etomidate, opioids, or their components;
2. Patients who participated in other clinical trials within 4 weeks of operation;
3. Severe cardiac insufficiency (acute heart failure, myocardial infarction, etc) or hemodynamic instability;
4. Patients with uncontrolled or poorly controlled hypertension;
5. Abnormal liver and kidney function: ALT and/or exceed 1.5 times of the upper limit of medical reference value; creatinine exceeds the upper limit of medical reference value;
6. Patients with myasthenia gravis or psychiatric disorders (schizophrenia, mania, bipolar disorder, cognitive dysfunction, etc.);
7. Patients with a history of alcohol abuse or addicted to opioids or benzodiazepine; and
8. Patients with expected difficult airway (e.g., thyromental distance ≤4 cm or Mallampati scores of 3 or 4).

##### Drop-out criteria

1. Participant’s request to withdraw the study;

2. Occurrence of serious complications or adverse events during the anesthesia;

3. Difficult intubation; and

4. Other reasons that enable the principal researcher to decide to terminate the study.

### Implementation

After all the participants have finished the preoperative evaluation and screening, participants will be informed of their randomized allocation. Eligibility evaluation and enrolment will be conducted by one independent investigator, while randomization will be finished by another investigator.

### Interventions

All patients will routinely fast for 8h before surgery. After the patient enters the operating room, standard anesthesia monitoring including electrocardiogram (ECG), noninvasive blood pressure (NIBP), peripheral oxygen saturation (SPO_2_), and bispectral index (BIS) will be routinely monitored. A 20-gauge intravenous cannula will be placed for Ringer lactate (10 ml/kg) infusion. Invasive arterial blood pressure (IBP) will be continuously monitored via insertion of a catheter into the left radial artery under local infiltration anesthesia with lidocaine. After preoxygenation, patients in Group E will be induced with etomidate (0.3 mg/kg), Group LR will be induced with remimazolam (0.2 mg/kg), and Group HR will be induced with remimazolam (0.3 mg/kg). Then, sufentanil (0.5 μg/kg) will be injected in all groups. After that, all patients will be injected with rocuronium (0.6 mg/kg) for muscle relaxation when loss of consciousness. Tracheal intubation will be performed two minutes after rocuronium administration.

Systolic blood pressure (SBP), diastolic blood pressure (DBP), mean arterial pressure (MAP), and heart rate (HR) will be measured and recorded at baseline (T0), 1 min after the induction of anesthesia (T1), 3 min after the induction of anesthesia (T2), immediately at tracheal intubation (T3), 1 min after tracheal intubation (T4), and 5 min after tracheal intubation (T5). Laryngoscopy and anesthesia administration will be performed by an anesthesiologist who is blinded to the study groups. The duration of laryngoscopy and intubation are also recorded for each patient.

Hypotension is considered when MAP falls below 60 mmHg or decreases to less than 20% of baseline during induction, and 50 μg norepinephrine will be given intravenously as needed. When HR decreases below 45 beats per min (bpm), it is considered severe bradycardia and 0.25 mg atropine or more will be administrated by intravenous injection. If the MAP rises above 120 mmHg, nitroglycerin will be given, and once HR rises above 120 bpm, esmolol will be given. The incidence of adverse cardiovascular events (hypotension, severe bradycardia, hypertension, tachycardia, and arrhythmia) and the cumulative doses of vasoactive drugs will be recorded.

### Outcomes

#### Primary outcome

The primary outcome of this study are the resultant changes in hemodynamic parameters: a) ▴MAP, defined as the difference of maximum or minimum MAP to baseline; b)▴HR, defined as the difference of maximum or minimum HR to baseline.

#### Secondary outcomes

The secondary outcomes include the following:

1. The incidence of adverse cardiovascular events (hypotension, severe bradycardia, hypertension, tachycardia, and arrhythmia).
2. The cumulative doses of vasoactive drugs used per patient.
3. Hemodynamic parameters (HR, MAP, SBP, DBP), BIS index, lactic acid, and glucose values
4. Incidence and degree of injection pain is measured using a 4-point graded scale: 0 = no pain, 1 = verbal complaint of pain, 2 = withdrawal of the arm, 3 = both verbal complaint and withdrawal of the arm.
5. Incidence and degree of myoclonus is recorded as follows: 0 = no myoclonic movements, 1 = minor myoclonic movements, 2 = moderate myoclonic movements, 3 = major myoclonic movements [16].

### Quality control

The principal investigator, study coordinator, and Office of Scientific Research at the Affiliated Hospital of Guizhou Medical University are jointly responsible for all aspects of the study protocol and amendments. Data collection and follow-up will be performed by two dedicated research residents. Designated trial monitors will periodically review all investigational data for accuracy and completeness to ensure protocol compliance.

### Data collection

Each participant’s characteristics, including age, sex, and body weight, as well as ASA grade, type and duration of surgery, vital signs, cumulative doses of vasoactive drugs, and the incidence of adverse events during induction, will be recorded in a designed data form by an investigator who is blinded to this study.

### Sample size

The sample size calculation was based on the primary outcome, the fluctuations in hemodynamic parameters (▴MAP). Based on our pilot data and relevant literature, assuming a type I error protection of 0.05 and a power of 0.90, 31 patients in each group are required in this study. Considering an estimated 20% dropout rate, 39 patients in each group will be included in this study.

### Statistical analysis

Statistical analysis will be performed by SPSS 22.0 software (SPSS Inc, Chicago, IL, USA). Data distribution will be tested for normality using the Shapiro-Wilk test. Continuous data will be presented as mean [standard deviation (SD)] when normally distributed or median (interquartile range [IQR]) when not normally distributed; Numerical variables will be compared using one-way analysis of variance (ANOVA) or repeated-measures ANOVA or Kruskal-Wallis tests after checking the normal distribution. Post hoc tests will be performed using a Bonferroni method. Categorical variables will be compared using a Fisher’s exact test or chi-square test, as appropriate. A *p* value < 0.05 is considered statistically significant.

### Auditing

In order to review and verify the development of the study throughout the trial period, the research team will hold a weekly meeting and submit a progress report every month.

### Protocol amendments

If necessary, decisions on protocol amendment will be made by the research team and authorized by the ethics committee.

### Dissemination

The database and related materials will be available accessible for the study staff and researchers of the project. Results will be published in open-access peer-reviewed journals within 12 months of completion of the trial.

## Discussion

Patients with cardiac disease often have different degrees of cardiac functional impairment and almost all anesthetic drugs possess capacity to suppress the myocardium and induce vasodilatation, therefore, these patients are prone to developing hypotension during anesthesia induction [17]. Post-induction hypotension is a threatening complication and is associated with increased morbidity and mortality. Considering the importance and impact of hemodynamic changes on complications and outcomes, anesthesiologists should comprehensively consider the effectiveness and safety when selecting anesthetics agents. An ideal induction agent would be one that is easy to administer, has rapid onset and rapid offset, and few adverse effects. However, such an agent does not exist.

Etomidate is a well-known hypnotic agent routinely used for induction of anesthesia [18]. It was previously considered to be superior compared to other anesthetic agents (such as propofol, midazolam, thiopental) for patients with hemodynamic instability owing to its lower inhibition effect on the cardiovascular system [19–21]. However, etomidate may lead to adrenal cortical function inhibition, nausea and vomiting, injection pain, and myoclonic movement, which are some undesirable adverse complications [22, 23]. Therefore, there is a need for anesthetic agents that have a rapid onset of action, short recovery time, and stable cardiovascular profile.

Remimazolam tosilate is an ultra-short-acting GABA-A receptor agonist [24]; a growing number of studies have shown that remimazolam display stable peri-induction hemodynamics, rapid onset, and rapid elimination [25–27], indicating that remimazolam may be a safe agent for patients with unstable pre-operative hemodynamics. A recent study showed that remimazolam was superior to propofol during anesthesia induction in patients undergoing valve replacement surgery [28]. Furthermore, Saito et al. [29] also reported that remimazolam could be used safely in induction and maintenance of anesthesia for cardiac surgery with cardiopulmonary bypass (CPB). Considering the advantages of remimazolam as mentioned above, remimazolam has the potential to be a better choice during induction and maintenance of anesthesia for cardiac surgery. Currently, there are few studies on the effect of remimazolam on hemodynamics during anesthesia induction in cardiac surgery. To the best of our knowledge, this is the first study comparing remimazolam tosilate and etomidate on hemodynamics during anesthesia induction in patients with cardiac disease. Thus, the aim of the study is to compare the effect of remimazolam tosilate and etomidate for anesthesia induction on hemodynamics in patients undergoing valve replacement surgery. This study will provide new clinical practice data for evaluating the effectiveness and disadvantages of remimazolam in the field of cardiac anesthesia.

### Public and patient involvement

There was no patient or public involvement in the design of the protocol. However, a patient and public involvement group has been assembled and will be consulted during dissemination of the findings.

### Trial status

The trial is currently in the recruitment phase. Trial completion is expected by December 2023.

## Data Availability

The database and related materials will be available accessible for the study staff and researchers of the project.

## Abbreviations

MAP: Mean arterial pressure
HR: Heart rate
GABA_A_: γ-Aminobutyric acid subtype A
ASA: American Society of Anesthesiologists
NIBP: Noninvasive blood pressure
SPO_2_: Peripheral oxygen saturation
BIS: Bispectral index
IBP: Invasive arterial blood pressure
SBP: Systolic blood pressure
DBP: Diastolic blood pressure
CPB: Cardiopulmonary bypass
